# A Role for Hemopexin in Oligodendrocyte Differentiation and Myelin Formation

**DOI:** 10.1371/journal.pone.0020173

**Published:** 2011-05-25

**Authors:** Noemi Morello, Federico Tommaso Bianchi, Paola Marmiroli, Elisabetta Tonoli, Virginia Rodriguez Menendez, Lorenzo Silengo, Guido Cavaletti, Alessandro Vercelli, Fiorella Altruda, Emanuela Tolosano

**Affiliations:** 1 Molecular Biotechnology Center, University of Turin, Turin, Italy; 2 Department of Neuroscience and Biomedical Technologies, University of Milan Bicocca, Monza, Italy; 3 Neuroscience Institute of Turin, Department of Anatomy, Pharmacology and Forensic Medicine, University of Turin, Turin, Italy; Universidade Federal do Rio de Janeiro, Brazil

## Abstract

Myelin formation and maintenance are crucial for the proper function of the CNS and are orchestrated by a plethora of factors including growth factors, extracellular matrix components, metalloproteases and protease inhibitors. Hemopexin (Hx) is a plasma protein with high heme binding affinity, which is also locally produced in the CNS by ependymal cells, neurons and glial cells. We have recently reported that oligodendrocytes (OLs) are the type of cells in the brain that are most susceptible to lack of Hx, as the number of iron-overloaded OLs increases in Hx-null brain, leading to oxidative tissue damage. In the current study, we found that the expression of the Myelin Basic Protein along with the density of myelinated fibers in the basal ganglia and in the motor and somatosensory cortex of Hx-null mice were strongly reduced starting at 2 months and progressively decreased with age. Myelin abnormalities were confirmed by electron microscopy and, at the functional level, resulted in the inability of Hx-null mice to perform efficiently on the Rotarod. It is likely that the poor myelination in the brain of Hx-null mice was a consequence of defective maturation of OLs as we demonstrated that the number of mature OLs was significantly reduced in mutant mice whereas that of precursor cells was normal. Finally, *in vitro* experiments showed that Hx promotes OL differentiation. Thus, Hx may be considered a novel OL differentiation factor and the modulation of its expression in CNS may be an important factor in the pathogenesis of human neurodegenerative disorders.

## Introduction

Myelin formation in the brain occurs predominantly postnatally in well-regulated steps during which oligodendrocytes (OLs) mature and extend processes to contact and enwrap axons [1], [2], [3], [4], [5], [6], [7], [8]. Myelin formation is critical for the proper function and maintenance of axons [9] and, on the other hand, the survival of OLs depends on their interactions with axons [10]. Myelin loss thus results in pathological conditions, such as multiple sclerosis in which demyelination and concurrent axonal loss lead to significant disabilities. Thus, finding the factors that can promote myelin formation is of major relevance for the physiological organization and function of the CNS.

Hemopexin (Hx) is an acute phase plasma glycoprotein with high heme binding affinity (*K*_d_<10^−9^ M) [11] mainly produced by the liver and, to a lesser extent, by several other extrahepatic sites. In particular, Hx protein was found in neurons and astrocytes of cerebral cortex, cerebellum, striatum and hippocampus [12], in ganglionic and photoreceptor cells of the retina, in Schwann cells of the peripheral nervous system and in kidney mesangial cells [13], [14], [15], [16]. Moreover, by using mice bearing a knock-in allele expressing beta-galactosidase instead of Hx, we demonstrated that in the CNS, Hx is produced by ependymal cells lining the ventricular system and by hippocampal neurons [17].

Hepatocyte-derived Hx is released into the plasma where it binds heme and delivers it back to the liver [18]. In this way, Hx acts as a protective protein against free heme-mediated oxidative stress, which contributes to iron homeostasis through the recycling of heme iron [19], [20]. On the other hand, extra-hepatic production of Hx has been proposed to have a protective role following local tissue damage [11].

Little is known about the role of Hx in the CNS, with the only data coming from the analysis of Hx^−/−^ mice. These animals are more susceptible to ischemic stroke than wild-type controls [12]. Moreover, they have a higher number of iron-loaded OLs compared to age-matched wild-type mice, which is not associated to an increase in ferritin expression resulting in oxidative stress [17]. These data suggest that OLs are particularly sensitive to the lack of Hx and, together with the notion that iron is an essential factor in OL biology [21], prompted us to investigate the myelination state of Hx^−/−^ mice.

We found a reduced myelination in Hx^−/−^ mice compared to age-matched wild-type controls, resulting in motor dysfunction. Hypomyelination is due to a defective differentiation of OLs associated to a progressive impairment of their function with aging. We provide data highlighting a double role for Hx in OL biology, as a “differentiation factor” at early stages of life and as a “maintenance factor” for the later stages.

## Materials and Methods

### Animals

Hx^−/−^ mice were generated in our laboratory as previously described [22]. Hx^−/−^ and wild-type mice used for experiments were in the 129Sv genetic background and were maintained on a standard diet. All procedures involving the use of live animals were performed under the supervision of a licensed veterinarian, according to guidelines specified by the Italian Ministry of Health (DDL 116/92) and were approved by the Ethics Committee of the Molecular Biotechnology Center (University of Torino, Italy).

### Histology

Animals were anaesthetised with Avertin (2,2,2-tribromoethanol; Sigma-Aldrich, Milano, Italy) at a dose of 2 mg/kg body weight and transcardially perfused with 0.1 M phosphate-buffered saline (PBS), pH 7.2 followed by fixative solution (4% paraformaldehyde in PBS). Brains were removed and post-fixed in the same fixative for 3 h at 4°C, washed in PBS, cryoprotected by immersion in 30% sucrose in PBS overnight, embedded and frozen in cryostat medium (Bio-Optica, Milano, Italy). Brains were cut in coronal 50 µm-thick, free-floating sections which were stored in PBS at 4°C for Black-Gold analysis or collected in a cryoprotectant solution (30% ethylene glycol and 25% glycerol in PBS) at −20°C for immunohistochemistry.

### Black-Gold myelin staining

Tissue sections were mounted onto gelatin-coated slides and air dried at 60 °C for 30 min on a slide warmer. Slides were then transferred to pre-warmed 0.2% Black-Gold solution dissolved in 0.9% saline vehicle, for 15 min at 60 °C. At this time, the extent of the impregnation was monitored microscopically every 2–3 min until complete myelin impregnation was observed, i.e. when the finest myelinated fibers were stained. To intensify the staining, slides were incubated in pre-warmed 0.2% potassium tetrachloroaurate (III) in saline, for 15 min at 60°C. Next, slides were rinsed for about 2 min in distilled water, fixed in a 2% sodium thiosulfate solution for 3 min and rinsed in tap water for 15 min. Sections were finally dehydrated and mounted with DPX (BDH Laboratory Supplies, Leicester, United Kingdom). Three Hx^−/−^ and three wild-type mice were used for each age-point, i.e. two, six and twelve months.

### Immunohistochemistry

Tissue sections were washed in PBS, transferred to gelatinized slides, air dried at 37°C and analyzed with the following antibodies: mouse monoclonal CC1 antibody (also called APC, Adenomatus Polyposis Coli; 1∶25; Calbiochem, Beeston Nottingham, United Kingdom), rabbit polyclonal anti-glial fibrillary acid protein (GFAP) antibody (1∶500, Dako Cytomation, Milano, Italy) and rat monoclonal anti-CD140a (also called PDGFRα, 1∶500, BD Biosciences, Erembodegem, Belgium). Briefly, tissue sections were rehydrated in PBS, incubated in 0.3% hydrogen peroxide in PBS (RT) for 20 min, treated with 0.3% Triton-X-100 in Tris-buffered saline (TBS) for 30 min and saturated with blocking buffer (3% milk, 10% normal swine serum in TBS) for 1 h, followed by antibody incubation at 4° C overnight. The following biotinylated secondary antibodies were used: swine anti-rabbit IgG, rabbit anti-rat IgG and rabbit anti-mouse IgG (DakoCytomation). Immunoreactivity was enhanced with Elite ABC system (DakoCytomation) and developed with DAB. Sections were transferred to gelatinized slides, rinsed in PBS, dehydrated and mounted in DPX (BDH Laboratory Supplies). To visualize mature OLs a protocol was developed to combine CC1 and GFAP immunohistochemistry. Sections were processed for the first primary antibody (mouse anti-CC1) using MOM kit (Vector Laboratories) and DAB developing method; the second primary antibody (rabbit anti-GFAP) was processed normally as previously described and developed with Vector SG substrate kit for peroxidase (Vector Laboratories, DBA ITALIA, Segrate, MI, Italy). Quantification of positive cells was performed with a computer-microscope system equipped with the NeuroLucida program (Microbrightfield, Inc., Williston, VT, USA). Positive cells were counted on 50 µm-thick serial sections obtained every 0.6 mm through the corpus callosum and the cortex region. Three Hx^−/−^ and three wild-type mice were used for each age-point, i.e. postnatal day 10 (P10) and 20 (P20).

### Western blotting analysis

Freshly dissected brain cortex and basal ganglia were washed and stored at −80 °C. Frozen samples were powdered with a pestle in the constant presence of liquid nitrogen and dissolved in 50 mM HEPES (N-2-hydroxyethylpiperazine-N-2-ethanesulfonic acid), 50 mM NaCl, 5 mM EDTA (ethylenediaminetetraacetic acid) with protease inhibitors (aprotinin, leupeptin, pepstatin; Sigma-Aldrich). Fifty µg of total protein extracts were separated on SDS-PAGE and analysed by Western blotting according to standard protocol using a rabbit polyclonal anti-Myelin Basic Protein (MBP) antibody (Immunological sciences, Rome, Italy) and a rabbit polyclonal anti-Actin antibody (Santa Cruz Biotechnology, Heidelberg, Germany). The intensities of bands were quantified with the Bio-Rad Gel Doc system using Quantity One software. Three Hx^-/-^ and three wild-type mice were used for each age-point, i.e. two and twelve months.

### Electron Microscopy (EM)

Twelve month-old Hx^−/−^ and wild-type mice were killed with an overdose of anesthetic and perfused transcardially with a washing solution (phosphate buffer – PB – 0.12 M, pH 7.4) followed by the fixative (4% paraformaldehyde, 2% glutaraldehyde in 0.12 M PB). Brains were removed, dissected, and the corpus callosum region post-fixed in the same fixative for 2 h before transferring to 0.12 M PB. Specimens were immersed in 1% OsO_4_ in cacodylate buffer, dehydrated in ethanol and embedded in epoxy resin. Ultrathin sections (60 nm) were obtained with an ultra microtome Ultracut E Reichert-Jung and then were stained with uranyl acetate and lead citrate for examination with a transmission electron microscope CM 10 Philips (FEI, Eindhoven, Netherlands). Randomly selected EM images were analyzed using the Image J 1.37v software (National Institute of Health, Bethesda, MD, USA). Five animals of each genotype were used.

### Behavioural testing: Rotarod

The motor coordination was examined by using an accelerating rotarod test (model 760; Ugo Basile, Varese, Italy). The rotating rod underwent linear acceleration from 4 to 32 rpm over the first 5 min of the trial. The rotarod test was performed by placing a mouse on a rotating treadmill drum (3 cm diameter) and measuring the time period for which each animal was able to maintain its balance on the treadmill. After two min training runs, the mice underwent testing three times for a maximum of 300 sec, and the mean latency to fall off the treadmill was recorded. Mice were analyzed from two to twelve months, two test sessions/month. Sixteen animals of each genotype were used.

### Isolation and culture of oligodendrocyte precursor cells

Primary OL cultures were prepared from forebrains of 3 to 4-day-old rat pups using a differential detachment method. Briefly, forebrains free of meninges were digested with Hanks Balanced Salt Solution (HBSS) containing 0.01% trypsin, and mechanically dissociated in DMEM 20S (Dulbecco's modified Eagles medium High Glucose (DMEM) with 20% fetal bovine serum, 2 mM L-glutamine, and 100 U/mL penicillin, 100 µg/mL streptomycin, 2.5 µg/mL Fungizone e 1 mM Na-piruvate). Dissociated cells were plated onto poly-L-lysine coated 75 cm^2^ flasks (1 cerebrum per flask) and maintained 9 days at 37°C in a humid atmosphere of 5% CO_2_ and 95% air; medium was changed completely every two days. Then, oligodendrocyte precursor cells (OPCs) were isolated from the mixed glial cells by the method of McCarthy and DeVellis [23]. Briefly, following 1 h pre-shake to remove microglia, flasks were shaken overnight at 200 rpm to separate OPCs from the astrocyte layer. Cell suspensions were then plated onto uncoated Petri dishes for 1 h to further remove residual contaminating microglia/astrocytes. Resulting OPCs were plated onto poly-D,L-ornithine-coated 24-well titer at a density of 30000 cells/well, and maintained in Neurobasal medium supplemented with B27 (NBB27), 10 ng/ml PDGF and 10 ng/ml bFGF for 4 days to stimulate the propagation of OPCs. At this time cultures were assessed to be greater than 95% OPCs by immunocytochemistry, using antibodies specific to astrocytes and microglial cells. The medium was then changed with NBB27 without growth factors and cells were treated with Hx purified from human serum (100 *n*mol/L; Athens Research & Technology, Athens, GA, USA), or with heme–Hx complex (100 *n*mol/L; heme was purchased by Frontier Scientific Europe, Carnforth, Lancashire, UK) for 2 days and then analyzed by immunofluorescence to evaluate OPC differentiation. Both Hx and heme were negative for endotoxin contamination.

### Immunofluorescence

Cells were fixed with 4% paraformaldehyde in PBS, quenched with 50 mM ammonium chloride, permeabilized with 0.1% Triton-X-100 in PBS for 30 min, saturated with blocking buffer (3% normal bovine serum in PBS) for 1 h and incubated for 2 h with the following primary antibodies: rabbit polyclonal anti-glial fibrillary acid protein (GFAP) antibody (1∶500, Dako Cytomation, Milano, Italy), rabbit polyclonal anti-IBA1 (1∶1000, Wako Chemicals GmbH, Neuss, Germany), rat monoclonal anti-CD140a (1∶500, BD Biosciences), rabbit polyclonal anti-PDGFRα (1∶500, Abcam, Cambridge, UK), mouse monoclonal anti-CNPase (1∶200, Millipore S.p.A., Milan, Italy) rat monoclonal anti-MBP (1∶100, Abcam, Cambridge, UK). Cells were then incubated for 1 h with the appropriate Alexa Fluor-conjugated secondary antibodies (Invitrogen, Carlsbad, CA, USA) and nuclei were stained using DAPI.

Cells were counted and categorized into four stages of OL differentiation according to two criteria: i)morphology: stage I: OPC (mono/bipolar), stage II: pre-OL (multipolar, primary branched); stage III: immature OL (multipolar, secondary branched); stage IV: mature OL (secondary branched cells with membranous processes) [24]; ii)marker expression, stage I and II: PDGFRα positive, CNPase negative, stage III: PDGFRα negative, CNPase positive, stage IV: PDGFRα negative, CNPase and MBP positive.

### Statistical Analysis

Difference among groups in terms of MBP density and OL count were analyzed by Student's t-test, a P-value less than 0.05 was considered significant. For comparison between the histogram distributions, the Chi-square goodness-of-fit test was used, the level of significance adopted was *P*<0.05. The plot of g-ratio was analyzed with Wilcoxon test for nonparametric data. For rotarod analysis, the time to fall in the rotating rod test was analyzed by two-way analysis of variance (ANOVA). *In vitro* experiments were analyzed by paired t-test.

## Results

### Hx^-/-^ mice show altered myelin basic protein expression in brain

**Figure 1:**
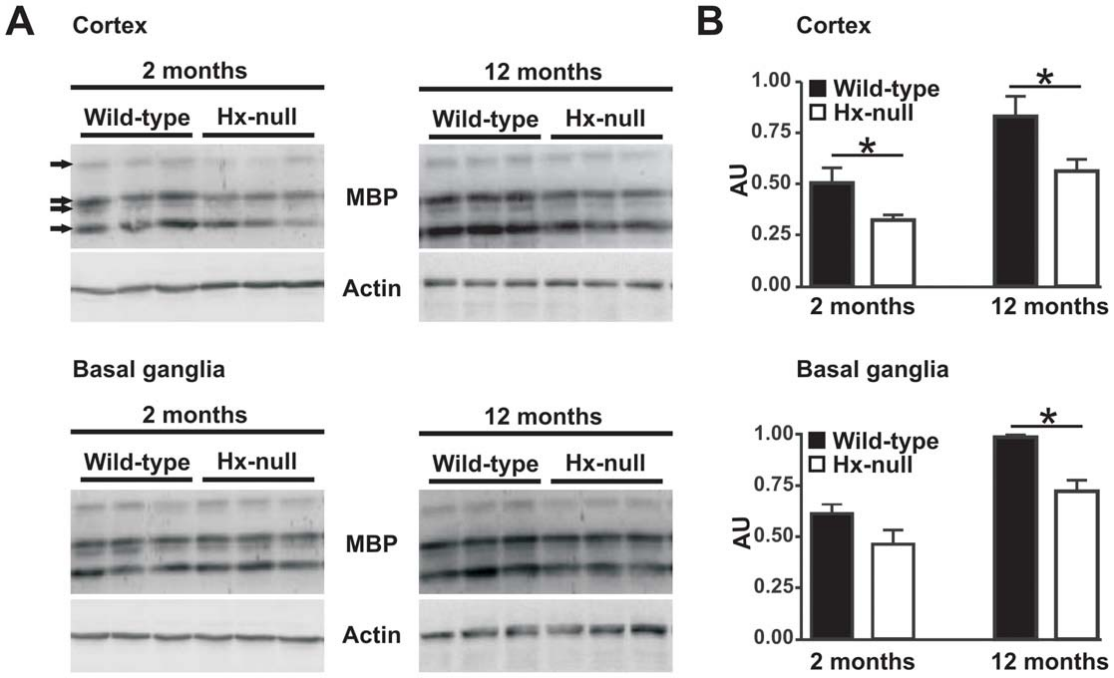
Reduction of MBP protein production in Hx^−/−^ brain. **A**) Western blot analysis of MBP expression in brain extracts of wild-type and Hx^−/−^ mice. Cerebral cortex and basal ganglia region lysates were analyzed at two and twelve months of age. Representative experiments are shown. **B)** Band intensities were measured by densitometry and normalized to actin expression (AU: Arbitrary Unit). The overall MBP production was obtained by summing the relative intensities of the four isoforms recognized by the antibody (indicated by arrows in scanned gels). Densitometry data represent mean ± SEM; n = 3 for each genotype. *  =  P<0.05. Results shown are representative of three independent experiments.

We have already demonstrated that OLs are more susceptible than other cell types to lack of Hx as they accumulate heme-derived iron [17]. As iron availability may affect the state of myelination [21], we posed the question whether iron load in OLs of Hx^−/−^ mice impairs their ability to form myelin and therefore analyzed the expression of MBP, one of the major myelin proteins [25], in two distinct regions of Hx^−/−^ mouse brains: cerebral cortex (motor and somatosensory areas) and basal ganglia, at two and twelve months of age. Immunoblot analysis on samples from two month-old mice showed a slight, but significant reduction in MBP content in the cortex of Hx^−/−^ mice compared to wild-type animals. In twelve month-old mice the reduction of MBP in Hx^−/−^ mice was more evident in both cerebral cortex and basal ganglia (Figure 1). The anti-MBP antibody revealed signals for all four bands of MBP, representing MBP splice variants and, in Hx^−/−^ mice, reduced expression affected all isoforms. Particularly, the less abundant 21.5 KDa isoform showed a reduction of about 60% in the cortex of 2 month-old mice, and of 40–50% in the cortex and basal ganglia of 12 month-old animals, whereas the other isoforms, 18.5, 17 and 14 KDa, showed a similar reduction of about 30–40% both in the cortex at 2 months and in the cortex and basal ganglia at 12 months of age (not shown).

### Hx^−/−^ mice show impaired myelination

**Figure 2:**
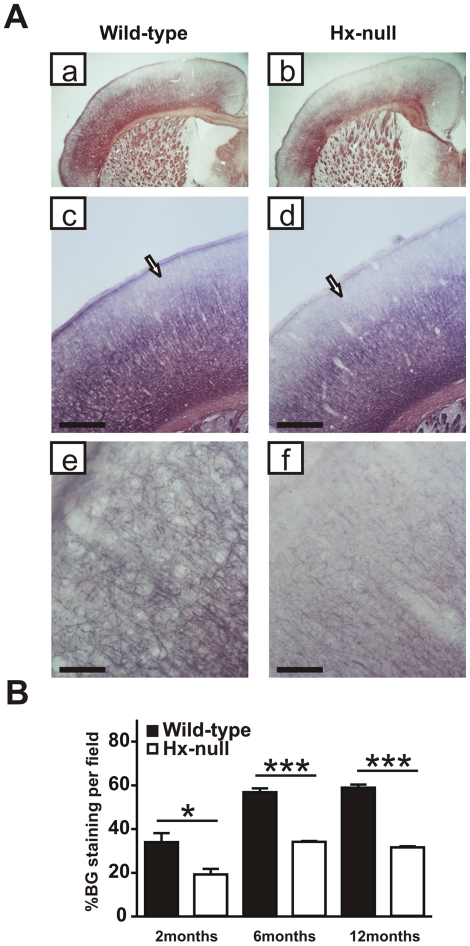
The cortex of Hx^−/−^ mice is hypomyelinated. **A**) Coronal sections of a wild-type and a Hx^−/−^ mouse at twelve months of age stained with Black-Gold reaction to detect myelinated fibers. Hx^−/−^ mouse shows reduced myelination in cerebral cortex compared to wild-type (a, b) and the hypomyelination mainly affects the supragranular layers in motor and somatosensory cortex (arrows in c, d). Higher magnification shows that in layer I of Hx^−/−^ mouse the staining is very weak compared to wild-type (e, f). Bar (c, d)  = 500 µm; Bar (e, f)  = 100 µm. **B**) Quantification of fiber density in motor cortical area, assessed at 2, 6 and 12 months of age, shows a severe reduction in Hx^−/−^ mice. Data represent mean ± SEM, n = 3 mice for each genotype. * = P<0.05, *** = P<0.001.

We investigated the structure of myelinated fibers in the cerebral cortex of 2, 6 and 12 month-old mice using the Black-Gold stain, which specifically labels myelin. Fiber systems such as the corpus callosum and the internal capsule are labeled in red, whereas individual axons appear dark black. Differences in myelin structure were already evident at two months of age, worsened at six months and persisted in 12 month-old mice (Figure 2). As shown in Figure 2A, brain sections of 12 month-old wild-type mice showed a diffuse and regular staining in all layers of the cortex, with a dense network of fibers, mostly horizontally oriented, in infragranular layers: from this network, dark black, thick axons originate, with a radial orientation, which ramify in the supragranular layers in a dense network of thin collaterals. In Hx^−/−^ brain sections, the density of the staining was clearly lower than in wild-type brain sections at low magnification. High-magnification images showed that in the supragranular cortical layers of the cortex, in particular layers I and II, in Hx^−/−^ mice, myelinated axons were less dense and thinner than in wild-type animals. This finding was corroborated by the observation of a reduced thickness of the axons radiating from infra- to supragranular layers. Affected areas include motor and somatosensory cortex. We quantified Black-Gold staining by densitometry in four consecutive sections in the motor cortical area: intensity of staining, indicative of the density of myelinated fibers, increased from 2 to 6 months of age and remained high at 12 months in both wild-type and Hx^−/−^ mice. In the latter we observed a 40–50% reduction in the density of myelinated fibers at all ages compared to wild-type controls (Figure 2B).

### Hx^−/−^ mice display altered myelin structure

**Figure 3:**
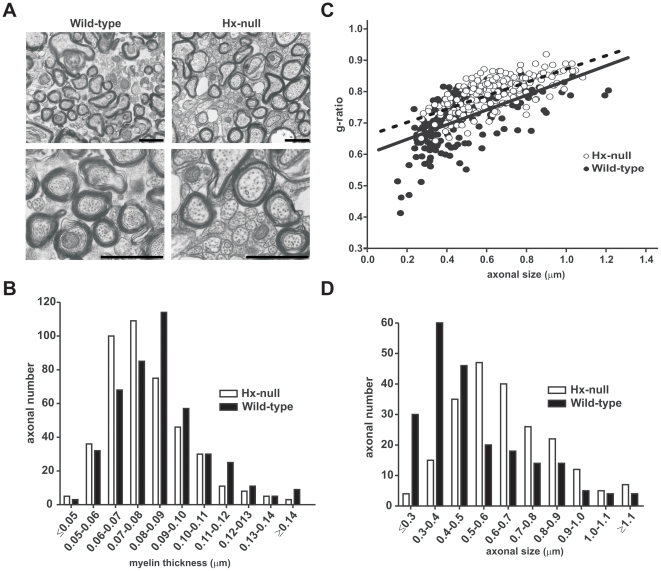
Alteration of myelin ultrastructure in the absence of Hx. EM analysis was performed on the corpus callosum of wild-type and Hx^−/−^ mice at twelve month of age. **A**) Electron micrographs show that in Hx^−/−^ mice the axons are hypomyelinated and the number of small myelinated axons is reduced in comparison to wild-types. Bar  = 1 µm. **B**) The distribution of myelin thickness in wild-type and Hx^−/−^ mice fibers demonstrated that myelin sheath was thicker in Hx^−/−^ fibers. P<0.0001. **C**) g-ratio scatter diagram in wild-type and Hx^−/−^ mice fibers. Elevated *g* ratio values were observed for all axons in Hx^−/−^ mice, indicating that impaired myelination affected axon of all sizes. P<0.001. **D**)The distribution of axonal size in wild-type and Hx^−/−^ mice fibers showed that Hx^−/−^ mice had bigger axons compared to controls. P<0.0001. n = 5 mice for each genotype.

To determine whether myelin structure as well as myelin density were compromised in Hx^−/−^ mice, we analyzed sagittal sections of corpus callosum from twelve month-old wild-type and Hx^−/−^ mice by EM. We did not find differences in the number of unmyelinated/myelinated fibers in the corpus callosum between Hx^−/−^ and wild-type mice (Table 1). Nevertheless, electron micrographs showed that the myelinated axons in Hx^−/−^ mice were severely hypomyelinated (Figure 3A, top). Higher magnification (Figure 3A, bottom) revealed that myelinated axons in Hx^−/−^ mice showed ultrastructural abnormalities. Indeed, unlike wild-type mice, Hx^−/−^ mice were characterized by the presence of numerous enlarged fibers with pale-stained cytoplasm in which neurofilaments were sparsely organized. Frequency distribution of myelin thickness (Figure 3B) revealed a shift towards lower values (Chi-square analysis, P<0.0001) in Hx^−/−^ mice fibers compared with wild-type mice, confirmed by an increase in the ratio of axon size to fiber diameter (g-ratio) in Hx^−/−^ mice compared with wild-type mice (Wilcoxon test, P<0.001) (Figure 3C). In addition, the g-ratio analysis revealed that hypomyelination affected axons of all size. Moreover, some myelinated fibers in Hx^−/−^ mice showed oligodendroglial cytoplasm between the compacted lamellas as well as between axon and the internal myelin layer. Interestingly, frequency distribution of axonal diameter (Figure 3D) showed a shift towards larger fibers in Hx^−/−^ mice compared with wild-type mice (Chi-square test, P<0.0001).

**Table 1:** Axon density in corpus callosum of wild-type and Hx^−/−^ mice.

Mice	Average No. of myelinated axons/field	Average No. of unmyelinated axons/field	Average No. of axons/field	Percentage unmyelinated axons
Wild-type	36.06±4.15	25.00±4.11	61.06±6.92	40.95
Hx^−/−^	33.90±2.91	25.50±5.02	59.40±5.50	42.93

The number of myelinated (axons containing compact myelin) and unmyelinated axons were counted in a 30 µm^2^ area from electron micrographs of corpus callosum sections. There were no changes in axon density between wild-type and Hx^−/−^ mice. Values are the average number of axons per field ± SD. Wild-type, n = 3; Hx^−/−^, n = 3.

### Hx^−/−^ mice exhibit motor dysfunction

**Figure 4:**
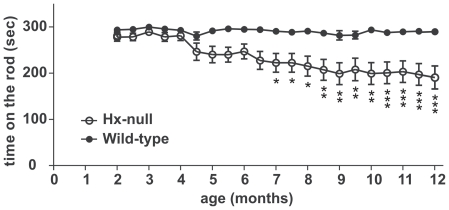
Motor dysfunction in Hx^−/−^ mice. Accelerated Rotarod tests were performed every fifteen days from two to twelve months of age. The mean time score in which the mice walked in synchrony with the rod was recorded. Each test consisted of three consecutive trials. Hx^−/−^ mice showed significant motor impairment starting from four months of age and increasing with age. Data represent mean ± SEM, n = 16 mice for each genotype. * =  P<0.05, ** = P<0.01, *** = P<0.001.

To assess whether hypomyelination in motor cortex of Hx^−/−^ mice had an effect on motor function, we tested motor coordination ability in these animals from 2 to 12 months of age, using the Rotarod test (Figure 4). The performance of wild-type mice was unchanged with increasing age, whereas in the Hx^−/−^ mice motor coordination deteriorated progressively by 4 months of age. The reduction in motor function in Hx^−/−^ mice became significant by seven months of age (mean score reduction of 23%) and worsened gradually until twelve months of age (mean score reduction of 34%).

### Hx^−/−^ mice have reduced number of mature OLs

**Figure 5:**
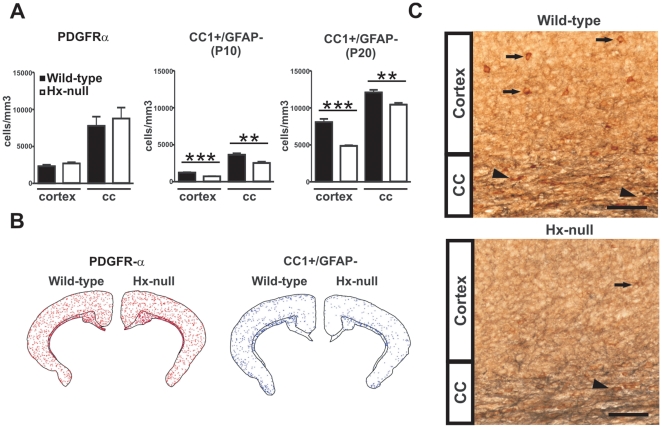
Impaired OL development in Hx^−/−^ mice. Brain sections of wild-type and Hx^−/−^ mice were immunoreacted to discriminate between OPCs and mature OLs and OPCs and OLs counted as reported in Materials and Methods. **A**) Quantification of PDGFRα-positive cells demonstrated similar numbers of OPCs in both cerebral cortex and corpus callosum in Hx^−/−^ and wild-type mice at P10. On the contrary, the number of CC1-positive, GFAP-negative mature OLs in Hx^−/−^ mice was strongly reduced compared to wild-type animals at P10 and P20. Data represent mean ± SEM, n = 3 mice for each genotype. **  = P<0.01, *** = P<0.001. B) Maps, obtained with Neurolucida/Neuroexplorer, of brain sections of PDGFRα- (left) and CC1- (right) positive cells, respectively, in Hx^−/−^ and wild-type mice at P10. Red =  OPCs, blue  =  mature OLs. Note the reduced number of mature OLs in the supragranular layer of cortex in Hx^−/−^ mice. C) Representative pictures of CC1/GFAP double staining for CC1 (brown) and GFAP (grey) in brain sections of a wild-type and a Hx^−/−^ mouse. The latter shows a strong reduction in the number of CC1 positive cells in the cortex (arrows) and in corpus callosum, CC, (arrow-heads) compared to wild-type animal. Bar  = 50 µm.

The deficit in myelin content of Hx^−/−^ mice might be the result of a reduction in the number of OLs or a reduction in the amount of myelin elaborated by each individual oligodendrocyte. To investigate this issue, we analyzed mature OL and OPC numbers in selected CNS areas, the cerebral somatosensory cortex and the corpus callosum, of Hx^−/−^ and control mice at P10 and P20. OPCs were identified as PDGFRα-labeled cells, and mature OLs as CC1-positive and GFAP-negative cells. At P10, we observed a significant 40% and 30% reduction in the number of mature OLs in Hx^−/−^ mice compared to wild-type animals in the cerebral cortex and corpus callosum, respectively, whereas OPC numbers were similar. The difference in the number of mature OLs persisted at P20 with a reduction of 40% and 15% in the cerebral cortex and corpus callosum, respectively (Figure 5A). Maps of positive cells in stained sections confirmed that Hx^−/−^ mice were characterized by fewer mature OLs in the corpus callosum and cerebral cortex compared to wild-type mice. In the somatosensory cortex, the reduction in the number of mature OLs mainly affected the supragranular layers (Figure 5B).

Because the density of OPCs is similar between wild-type and Hx^−/−^ mice, these data suggest that Hx is involved in regulating the differentiation and/or the survival of OLs.

### Hx promotes OPC differentiation *in vitro*

**Figure 6:**
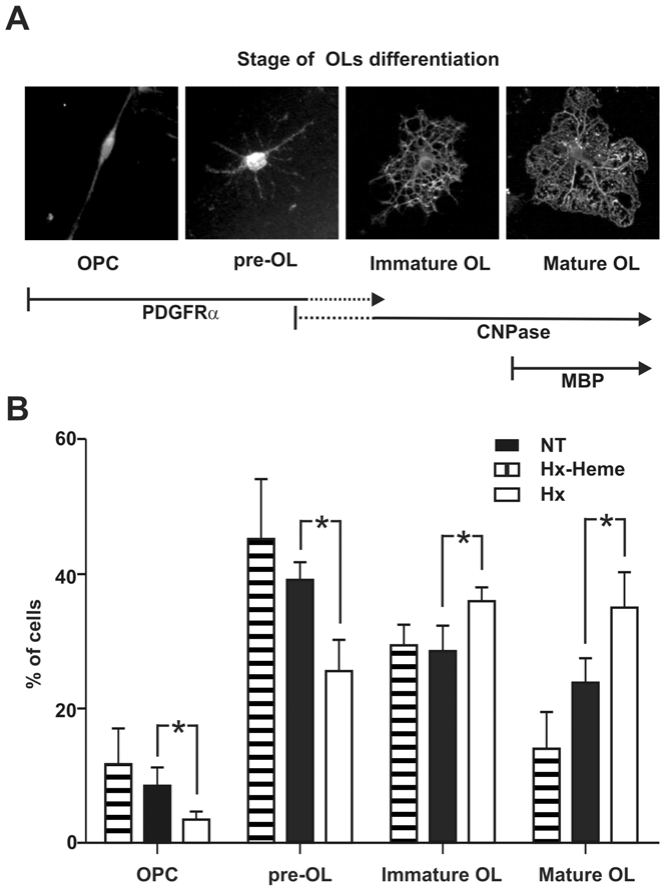
Hx promotes OL differentiation. OPCs were grown with or without Hx and the differentiation process was analyzed. **A**) Representative images showing the different developmental stages taken into consideration: stage I, OPCs (bipolar); stage II: pre-OL (primary branched); stage III: immature OL (secondary branched); stage IV: mature OL (secondary branched cells with membranous processes). Cells at stage I and II are PDGFRα positive, CNPase negative, cells at stage III are PDGFRα negative, CNPase positive and cells at stage IV are PDGFRα negative, CNPase and MBP positive. **B**) Kinetics of OL differentiation. Cells were cultured for 48 h in the absence (NT) or presence of Hx (Hx) or heme-Hx complex (Hx-heme), and the number of cells at each differentiation stage was counted as reported in Materials and Methods. Cells were scored by morphology and immunoreactivity to PDGFα and CNPase as shown in (A). Hx treatment accelerated the differentiation process whereas the heme-Hx complex was ineffective. *  =  P<0.05. Results shown are representative of three independent experiments.

As described in the previous paragraph Hx^−/−^ mice showed a deficit in mature OLs whereas OPC numbers were normal. Hx synthesis in the CNS starts in mice by P7 and progressively increases until adulthood and mainly occurs in ependymal cells lining the subventricular zone where OPCs arise (data not shown). These issues suggest that Hx may affect OPC differentiation. To test this hypothesis we analyzed the effect of Hx on OPC cultures: differentiation of OPCs was assessed on the basis of both morphological and immunological changes (Figure 6A).

After 48 h in the differentiation medium, in untreated OPC cultures we observed approximately an equal number of immature (PDGFα-positive, stage I–II) and more differentiated cells (PDGFα-negative, CNPase-positive, stage III–IV). In contrast, Hx treatment markedly accelerated the differentiation process resulting in a significant reduction of the number of immature cells in favour of an increase of mature OLs (30% of cells at stages I–II and 70% at stages III–IV) (Figure 6B). Moreover, Hx treatment produced an increase in the number of terminally differentiated myelin-forming OLs characterized by MBP expression (not shown). In agreement with the immunological changes, the presence of Hx in the differentiation medium produced an increase in the complexity of cell processes. In fact, accordingly to immunofluorescence data, in Hx-treated cultures we observed less cells with bipolar morphology (stage I) or primary branches (stage II) and a higher number of cells with secondary branches or membranous processes (stage III–IV).

Conversely, heme-bound Hx was not able to produce significant modifications in the morphology or in the immunological properties of OPCs (Figure 6B).

Taken together these data indicate that Hx has a pro-OL differentiation activity that is inhibited by interaction with heme.

## Discussion

In this work we have highlighted for the first time a role for Hx in OL development by demonstrating that the brains of Hx^−/−^ mice are hypomyelinated, likely due to a result of a defective OPC maturation. Moreover, we have shown that Hx is able to promote OL differentiation in culture.

Hypomyelination, demonstrated by reduced MBP expression and Black-Gold staining and by ultrastructural abnormalities, was already evident in 2 month-old Hx^−/−^ mice and worsened with aging. We reported that in Hx^−/−^ mice the less abundant MBP isoform of 21.5 KDa was more severely reduced than the other three. Interestingly, an isoform-specific reduction of MBP expression has already been observed in other knock-out models and has been correlated to functional differences among the isoforms [26], [27], [28]. Thus, it is possible to speculate that the lack of Hx affects the expression of specific populations of myelinating cells.

Our *in vitro* data showed that Hx treatment increased the number of cells with more mature morphologies (immature and mature OL) and reduced the number of cells displaying the immature morphology (OPC and preOL). This might occur through the activation of specific signalling pathways governing cell differentiation. Others have already reported that inhibition of the MAPK/ERK pathway generated an opposite result to what we obtained with Hx, i.e. a significant increase and decrease in the number of immature and mature cells respectively, associated to the appearance of a novel subpopulation of OL, positive for CNPase and MBP but without cellular processes, indicating that MAPK/ERK signalling is needed for oligodendroglial branching [29]. We did not observe this subpopulation in our cultures but only an increase in the number of more mature cells, that is, cells with secondary branches. This might indicate that Hx is able to potentiate the MAPK/ERK pathway, directly through the binding to a specific receptor or indirectly by modulating the activity of another ligand and/or receptor.

The phenotype of Hx^−/−^ mice shows striking similarities with that of mice lacking matrix metalloprotease (MMP)9 and/or MMP12, which are characterized by deficient myelination during development correlated to a decrease in mature OLs in spite of a normal precursor cell number [30]. This has been imputed to the ability of MMP9 and MMP12 to degrade specific substrates that inhibit OL differentiation, one of which being Insulin Growth Factor binding protein 6 (IGFBP-6) [30]. Interestingly, the kinetics of Hx expression in the CNS is very similar to that of MMP9, with both proteins starting to be produced by P7 and increasing steadily from P14 to P28, a temporal pattern that coincides with developmental myelination [31]. In addition, both Hx and MMPs are cleared from the extracellular environment by low-density lipoprotein receptor-related protein-1 (LRP-1) [32], [33], a scavenger receptor expressed in CNS by epithelial cells of choroid plexi, endothelial cells of microvessels, neurons, perivascular astrocytes, and microglial cells [34], [35], [36].

Furthermore, Hx and most MMPs share the so called “hemopexin domain”, a structural motif that is involved in MMPs' activation/inhibition, binding and cleavage of different substrates, localization and degradation [37]. Thus, we may speculate that during developmental myelination Hx modulates the activity of some proteases of the extracellular environment or of the cell membrane crucial for OL differentiation. In particular, our in vitro data showing that Hx is able to promote OL differentiation in serum-free media on minimally coated substrates, suggest that Hx activity may modulate some autocrine circuit essential for differentiation.

On the other hand, the fact that both Hx purified from human plasma and the recombinant protein show protease activity [38], [39], [40], [41], leaves open the possibility that Hx *per se* may degrade some critical substrate for OL differentiation.

The effect of Hx on OL differentiation in vitro is inhibited by binding of Hx to its ligand heme suggesting that the different Hx functions of promoting OL differentiation and scavenging free heme are mutually exclusive. It is possible that the binding of Hx with heme may mask some critical domain for OL differentiation. It is important to note that the concentration of Hx in the differentiation medium used in this study is very low (0.1 µM), comparable to that of MMPs analyzed in other works and in agreement with the amount of protein expected in brain parenchyma, whereas the heme scavenging function, mediated by plasma Hx, occurs at very high protein concentration (10–20 µM).

Interestingly, deficient myelination in MMP9^−/−^ and/or MMP12^−/−^ mice occurred transiently from P7 to P14 [30], whereas in Hx^−/−^ mice it worsened with age as we observed a reduced number of mature OL at P10 and P20 that correlated with reduced MBP expression at 2 months of age and impaired myelin deposition. The deficit in myelin deposition was already evident at 2 months of age and worsened from 2 to 6 months suggesting that Hx is not only involved in the early stages of OL differentiation, but also at later stages of myelination and in OL maintenance. Finally, the myelin deficit results in the impairment of motor function which was consistently evident by 4 months and progressively declined. The role of Hx in OL maintenance is likely due to its ability in preventing heme-mediated oxidative injury and/or in modulating iron accumulation in OLs [17]. Indeed, we have previously shown that adult Hx^−/−^ mice have a significant higher number of iron-overloaded OLs compared to wild-type animals and this results in the induction of markers of oxidative stress, the latter being likely responsible for the impairment of OLs function with age.

On the other hand, due to the crucial role of iron in myelination [21], it should also be taken into account the possibility that Hx may affect myelination by controlling heme-iron delivery to OLs. However free heme is a dispensable source of iron in physiologic condition becoming heme-iron highly available only under pathologic conditions associated to hemolysis. OLs obtain much of their iron in the form of inorganic ion via receptor-mediated uptake of H-ferritin [21]. Even though we have previously reported that both H- and L-ferritin levels were reduced in the brain of Hx−/− mice [17], that reduction was due to a decreased number of ferritin-positive cells limited to the cortical regions that now can be imputed to the deficit in OL cell number. Being iron available to a reduced number of cells, it likely accumulates in excess thus rendering OL more susceptible to oxidative damage.

Putting together this and our previous work, we conclude that the reduced number of OLs that reach maturity in Hx^−/−^ brains, due to the lack of a differentiation factor, are further exposed to a higher amount of iron which in turn worsen their viability and functional activity. In addition, Hx might limit oxidative damage by scavenging free heme eventually released by cerebral tissue, the latter function becoming much more important in hemolysis-associated pathologic conditions. Consistently, it has recently been reported that Hx^−/−^ mice are more susceptible to ischemic stroke and intracerebral hemorrhage [12], [42]. Moreover, Hx has been found to be strongly increased in the cerebrospinal fluid of patients suffering from Alzheimer's disease [43], further suggesting a potential protective role.

At the ultrastructural level, we found that in Hx^−/−^ mice there was a significant increase in axons bearing a thinner myelin sheath than normal in absence of differences in the number of unmyelinated/myelinated fibers. This is in concordance with the reduced number of OLs expected to myelinate a normal number of axons. The abnormalities in myelin deposition observed in Hx^−/−^ mice are likely expression of a disturbance in myelin compaction that again might be imputed to an effect of Hx on specific subpopulations of myelinating cells [44], [45]. Hx^−/−^ axons showed enlarged diameter and disorganized neurofilaments, typical of dystrophic axons: these alterations are likely to be the morphological substrates for motor dysfunction, since the organization of neurofilaments is fundamental for maintaining the three-dimensional array of axoplasm and the conduction properties of the axonal fiber [46].

We focused our functional analysis on motor behaviour, but our data indicate that the myelin deficit occurs throughout the cerebral cortex of Hx^−/−^ mice, thus suggesting that not only motor function, but also sensitive and cognitive functions may be affected by the lack of Hx. Moreover, the results of this work are in line with those previously reported on the expression of Hx after peripheral nerve injury [47], [48], [49]. In fact, Madore and co-workers demonstrated that, after sciatic nerve crush, Hx mRNA is expressed by Schwann cells, fibroblasts and invading blood macrophages: the protein accumulates in the extracellular matrix and its expression progressively declines during nerve regeneration, thus suggesting a role in nerve repair. We may now speculate that this role resides in the ability of Hx to also promote myelination in the PNS.

In conclusion, we have identified a novel factor important for developmental myelination. Our work sheds light on future strategies to repair myelin in the adult CNS after a demyelinating insult.
